# Development of *in vitro* and *in vivo* neutralization assays based on the pseudotyped H7N9 virus

**DOI:** 10.1038/s41598-018-26822-6

**Published:** 2018-05-31

**Authors:** Yabin Tian, Hui Zhao, Qiang Liu, Chuntao Zhang, Jianhui Nie, Weijing Huang, Changgui Li, Xuguang Li, Youchun Wang

**Affiliations:** 1 Division II of In Vitro Diagnostics for Infectious Diseases, National Institutes for Food and Drug Control, No. 2 Tiantanxili, Beijing, 100050 China; 2 Division of HIV/AIDS and Sexually-transmitted Virus Vaccines, National Institutes for Food and Drug Control, No. 2 Tiantanxili, Beijing, 100050 China; 3 Division of Respiratory Virus Vaccines, National Institutes for Food and Drug Control, No. 2 Tiantanxili, Beijing, 100050 China; 4 Centre for Biologics Evaluation, Biologics and Genetic Therapies Directorate, Health Canada, Ottawa, On, K1A 0K9 Canada

## Abstract

H7N9 viral infections pose a great threat to both animal and human health. This avian virus cannot be handled in level 2 biocontainment laboratories, substantially hindering evaluation of prophylactic vaccines and therapeutic agents. Here, we report a high-titer pseudoviral system with a bioluminescent reporter gene, enabling us to visually and quantitatively conduct analyses of virus replications in both tissue cultures and animals. For evaluation of immunogenicity of H7N9 vaccines, we developed an *in vitro* assay for neutralizing antibody measurement based on the pseudoviral system; results generated by the *in vitro* assay were found to be strongly correlated with those by either hemagglutination inhibition (HI) or micro-neutralization (MN) assay. Furthermore, we injected the viruses into Balb/c mice and observed dynamic distributions of the viruses in the animals, which provides an ideal imaging model for quantitative analyses of prophylactic and therapeutic monoclonal antibodies. Taken together, the pseudoviral systems reported here could be of great value for both *in vitro* and *in vivo* evaluations of vaccines and antiviral agents without the need of wild type H7N9 virus.

## Introduction

H7N9 has caused annual human infections with high fatality rate since it was first identified in humans in 2013^1^. According to the 2017 report by the World Health Organization, 1564 cases of H7N9 infection have been confirmed in humans, including at least 612 death cases^2^. Although only a few cases of human-to-human transmission were reported, the identification of multiple binding receptors has raised concerns about the pandemic potential of this virus^3,4^.

Numerous candidate vaccines and therapeutic antibodies are currently being evaluated^5–7^. Similar to clinical evaluation of seasonal influenza vaccines, *in vitro* assays such as hemagglutination inhibition (HI) and microneutralization (MN) assays are used to determine antibody titers in humans immunized with H7N9 vaccines; in preclinical studies, protections afforded by vaccines or antiviral agents were analyzed through monitoring survival rates or weight loss of the animals following challenges using wild type H7N9 viruses (wt H7N9). However, both *in vitro* assays and *in vivo* animal studies require the use of live H7N9 viruses, which could hinder research and development of vaccines and antivirals. To circumvent the need of live virus, pseudoviruses have been explored to detect specific antibodies against H1N1, H5N1 and H7N9^8–10^. H7N9 pseudovirus previously reported was based on the backbone pNL-4.3, but the titer of the virus was found to be very low, rendering it useless in animal studies. Here we succeeded in developing high-titer H7N9 pseudovirus and used them for *in vitro* and *in vivo* evaluation of vaccines and antibodies.

## Results

### Generation of pseudovirus and development of pseudovirus-based neutralizing assay (PBNA)

Previous reports revealed that the influenza pseudovirus based on lentiviral vector pNL4-3-Luc.R.E had a very low titer, and failed to infect animals. Recently, our laboratory developed a highly productive pseudovirus system by modifying SG3 HIV vector (pSG3.Δenv). This backbone plasmid system has been successfully used for generating various high-titer pseudovirus including in rabies^11^, Marburg^12^ and Ebola pseudoviruses^13^. In order to investigate whether this modified plasmid could be employed to improve the yield for H7N9 pseudovirus, we compared pSG3.Δenv-FlucΔnef with pNL4-3-Luc.R.E. We found the yield of pseudovirus based on the new plasmid was at least 100-fold higher than the previously reported system (Supplementary Fig. 1). Notably, both HA and NA are needed for high-titer pseudovirus production (Fig. S1). H7N9 pseudovirus generated in the new system was then used in all subsequent experiments.

**Figure 1:**
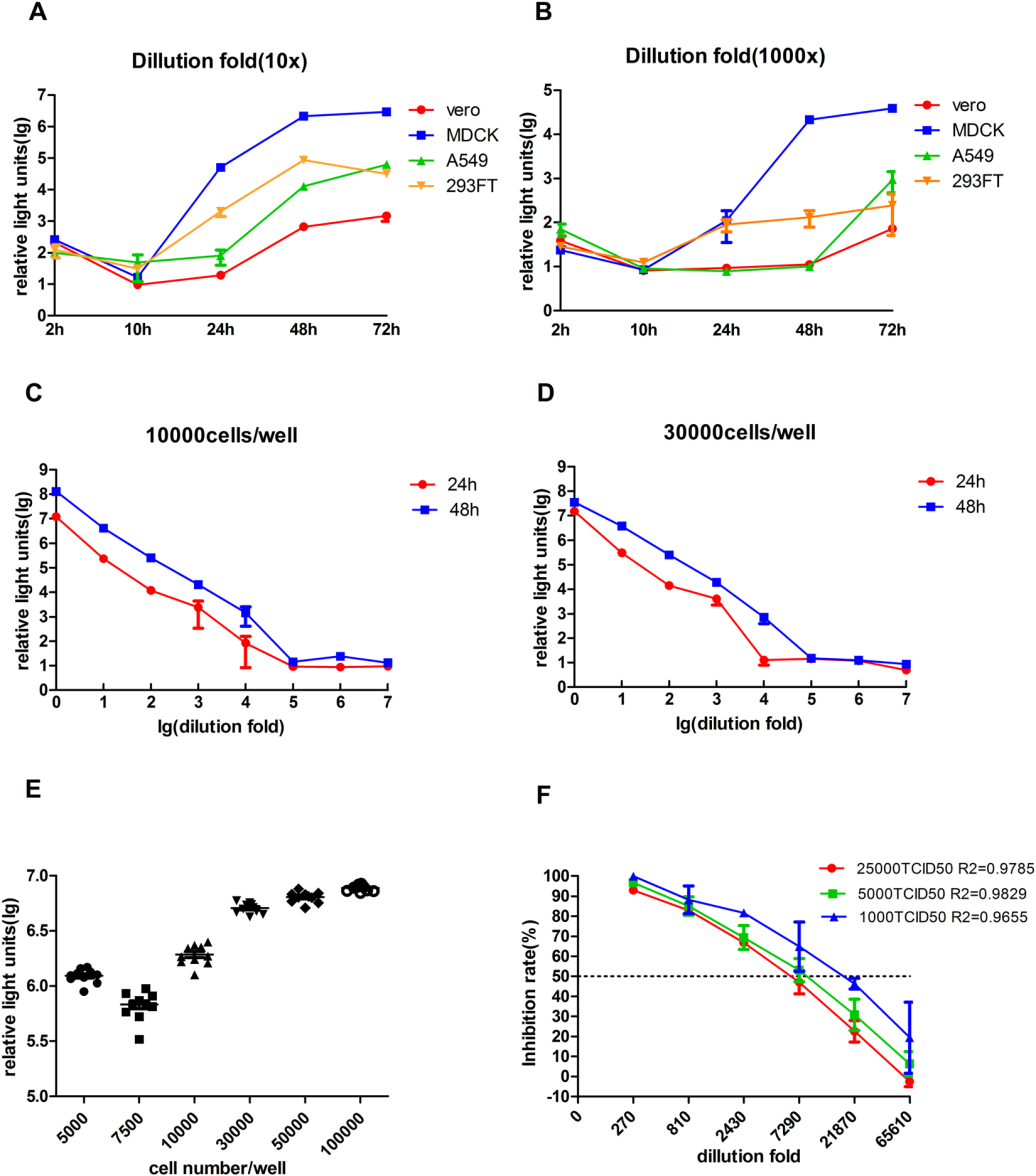
Development and optimization of PBNA. Panels (A,B) Selection of cell lines. The X-axis indicates the time (hours) after H7N9 pseudovirus infections while the Y-axis represents the RLU in the luciferase assay. Panel (A) 10000 TCID50 virus/30,000 cells; Panel (B) 100TCID50/30,000 cells. Panels (C,D) Determination of assay time points. The X-axis indicates serial dilutions of H7N9 inocula. Panel (C) 10000 cells/well. Panel (B) 30000 cells/well. Panels (E) Determination of MDCK cell density. MDCK cells were seeded from 5000 to 100000 cells in 96-wells plate and luciferase activity was detected at 48 h. Panel (F) Determination of H7N9 pseudovirus concentrations. Different virus dose were compared in neutralizing assay. Each data represents the mean luciferase activities of 3 replicates.

We next conducted an array of experiments to optimize PBNA. Of the four cell lines tested, MDCK cells were found to produce the highest levels of florescence activity (Fig. 1, panels A and B), with the virus yield at 48 hr being better than 24 hr after infection (Fig. 1C and D). Furthermore, we also investigated the number of cells to seed in each well, and found that 30000 cells/well was the optimal cell density (Fig. 1E). Finally, we determined the amount of virus used in PBNA, and found better correlation coefficient with 5000 TCID50 (R2 = 0.9829) than 25000 TCID50 (R2 = 0.9785) and 1000 TCID50 (R2 = 0.9655).

### Specificity and sensitivity of PBNA

**Figure 2:**
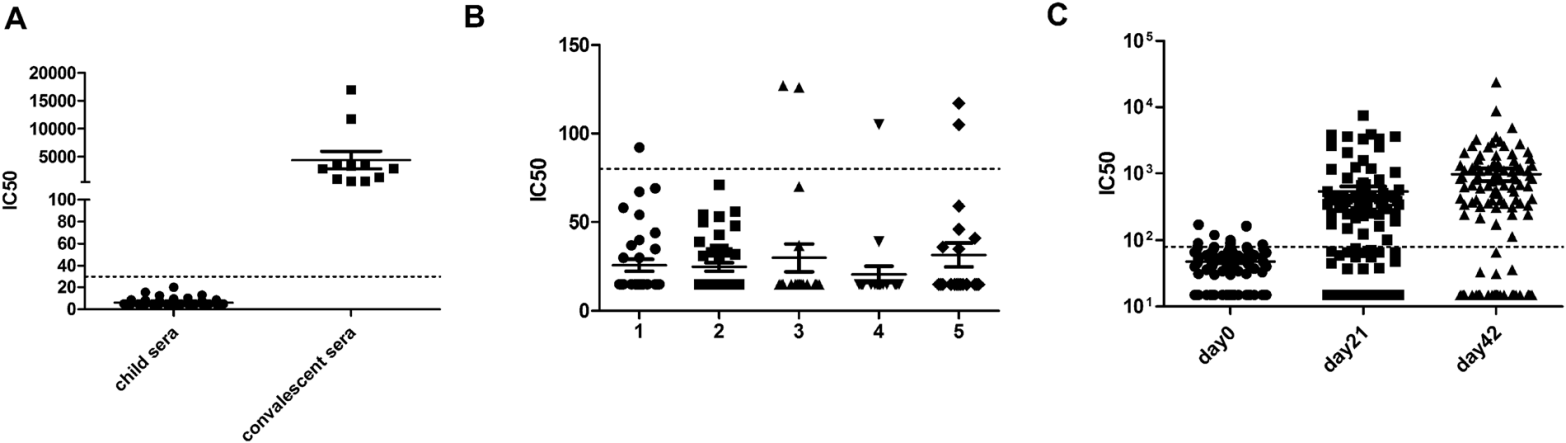
Evaluation of PBNA. Panel (A) Detection of pediatric sera and adult convalescent sera by PBNA. Panel (B) Detection of the sera obtained from various groups. Group 1~3 represents healthy participants in different age groups (0–20, 30–40 or 50–60 years old), Group 4 and 5 represent samples obtained from vaccinees before and after immunization with trivalent influenza vaccine. Panel (C) Detection of the sera obtained from H7N9 vaccinated individuals at the day 0, day 21 and day 42.

PBNA was assessed for its specificity and sensitivity using 75 negative serum samples from unexposed children and 11 serum samples from patients recovered from confirmed H7N9 infection. As expected, no H7N9 specific antibody was detected in the pediatric samples (IC50 < 30), whereas anti-H7N9 titers were found to range from 500 to 16000 in the convalescent sera (Fig. 2A). Moreover, sera from health adults were tested for the presence of H7N9 antibodies, and the titers in most samples were found to be less than 80 (Fig. 2B); it is of note that in these adults there were a few samples with titers ranging from 80 to 127, which is not surprising, given that adults have been exposed to various subtypes or strains of viruses, resulting in low levels of antibodies with cross-reactivity. 339 serum samples from the H7N9 vaccinated individuals were also tested. The titre of anti-H7N9 antibodies following vaccination significantly increased, whereas the titre before vaccination was similar to that observed in the health population (Fig. 2C). Taken together, the PBNA assay is a sensitive and specific assay for the determination of H7N9 antibodies in human subjects.

### Comparison of PBNA and conventional neutralization method

**Figure 3:**
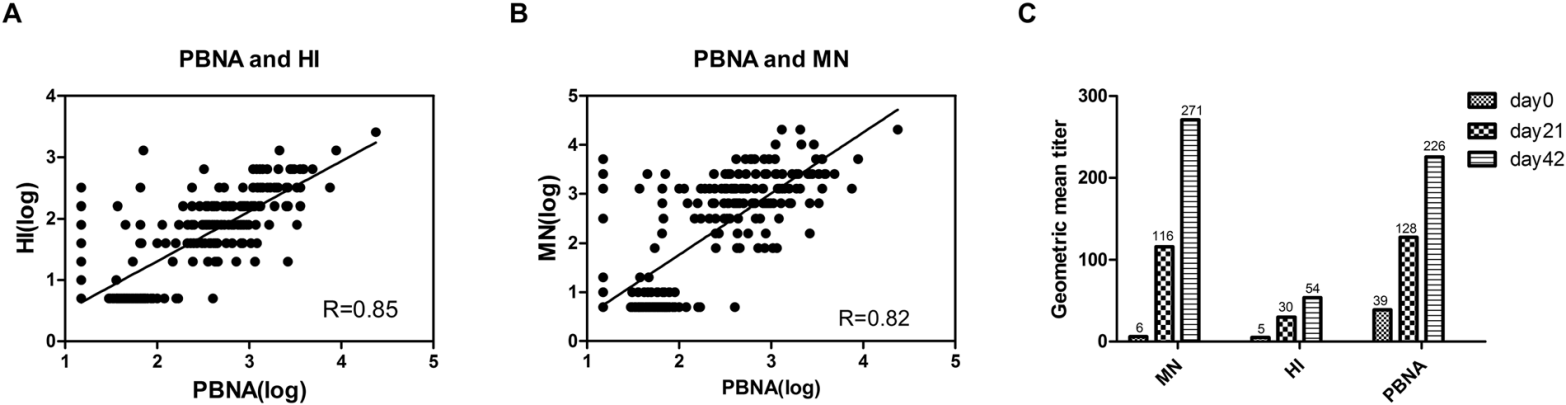
Comparison of PBNA with HI and MN. Panel (A) Comparison between PBNA and HI (R = 0.85, P < 0.0001, n = 339). Panel (B) Comparison between PBNA and MN (R = 0.82, P < 0.0001, n = 339). Pearson’s correlation analysis was used to assess the correlation between PBNA and HI or MN. Panel (C) Comparison of the geometric mean titers (GMT) obtained from MN, HI and PBNA.

We next set out to compare the correlation between PBNA and the traditional antibody assays, i.e., MN and HI. To this end, 339 serum samples above described from clinical trials were subjected to analysis using the three assays. The overall agreements between PBNA and the two traditional assays are good; specifically, the correlation coefficient between PBNA and HI was 0.85 (Fig. 3A), while the correlation coefficient between PBNA and MN was 0.82 (Fig. 3B); further statistical analysis revealed a highly significant correlation in antibody titers obtained by PBNA with those by HI or MN (p < 0.0001).

To further investigate the correlations between PBNA and traditional assays, we compared geometric mean titers (GMT) of H7N9 antibody collected at different time points after vaccination. As shown in Fig. 3C, the GMT values obtained by PBNA were closer to that by MN than HI at all time points. It is also clear that both PBNA and MN were more sensitive than HI as demonstrated by the five-fold higher GMT values (Supplementary Table 1). These results suggest that results by PBNA were more correlated with those generated by MN.

As PBNA appears to be in better agreement with MN, we further investigated the relationship between PBNA and MN in terms of sensitivity and specificity. The cutoff titer for MN is 1/80, while for PBNA the cutoff titer is 1/123. In terms of agreement with MN, the PBNA is 98% (95% CI: 0.96–1.00) and 92% (95% CI: 0.88–0.96) for sensitivity and specificity, respectively (κ = 0.89, Supplementary Table 2). Collectively, these data indicate that there is a strong correlation between PBNA and MN.

### Development and optimization of bioluminescent imaging mice model

**Figure 4:**
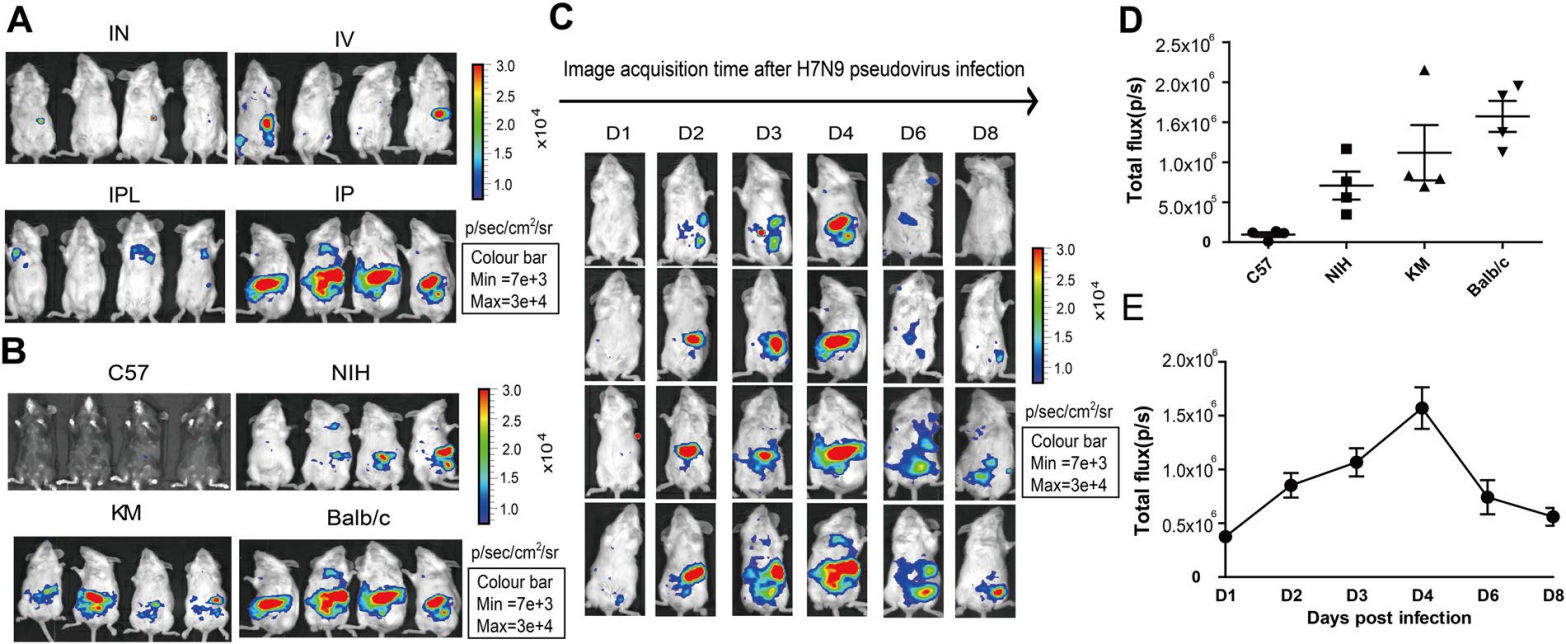
Development and optimization of bioluminescent imaging model. Panel (A) Imaging analysis of five- to six-week-old Balb/c mice inoculated with H7N9 pseudotype virus (5.62 × 10^6^ TCID_50_ per mouse) via intranasal (IN.), intravenous (IV.), intrapleural (IPL.), or intraperitoneal (IP.) routes (n = 4/group). Panel (B) Selection of mouse strains: different mouse strains infected intraperitoneally with 5.62 × 10^6^ TCID_50_ pseudovirus were imaged on day 4 (n = 4/group). Panel (C) Balb/c mice was imaged with intraperitoneally infected H7N9 pseudovirus (5.62 × 10^6^ TCID_50_) on day 1, 2, 3, 4, 6 or 8 (n = 4/group). Panel (D) Results of photon flux obtained with different mouse strains on day 4. All results are shown as mean ± s.e.m (n = 4/group). Panel (E) Values of photon flux from day 1 to 8 for Balb/c mice, with results shown as mean ± s.e.m (n = 4/group).

To assess whether the bioluminescent signal of H7N9 pseudovirus could be detected in mice, 5~6 week old Balb/c mice were used as they were known to be highly susceptible to H7N9 infection^14,15^. The animals were administered with the pseudovirus via different routes, followed by imaging analyses. As shown in Fig. 4A, strongest fluorescence signal was detected in mice infected with the pseudovirus intraperitoneally.

We next tested four different mouse strains (Fig. 4B) and observed dynamic biodistribution of pseudoviruses in these animals (Fig. 4C and Supplementary Fig. 2). The results showed that both Balb/c and Kunming (KM) mice produced strong fluorescence signals following i.p. injection (Fig. 4D). Specifically, after virus administration the signal increased over time and reached its peak on day 2 for KM and on day 4 for Balb/c (Fig. 4E and Fig. S2). We decided to choose Balb/c mice because they are readily available and well characterized inbred mouse strain. Therefore, day 4 was chosen for subsequent experiments. Finally, we found 5.62 × 10^6^ TCID_50_ per mouse was the ideal challenging dose in animal imaging analyses (Supplementary Fig. 3).

### Assessment of anti-H7N9 monoclonal antibody using *in vitro* and *in vivo* PBNA assays

**Figure 5:**
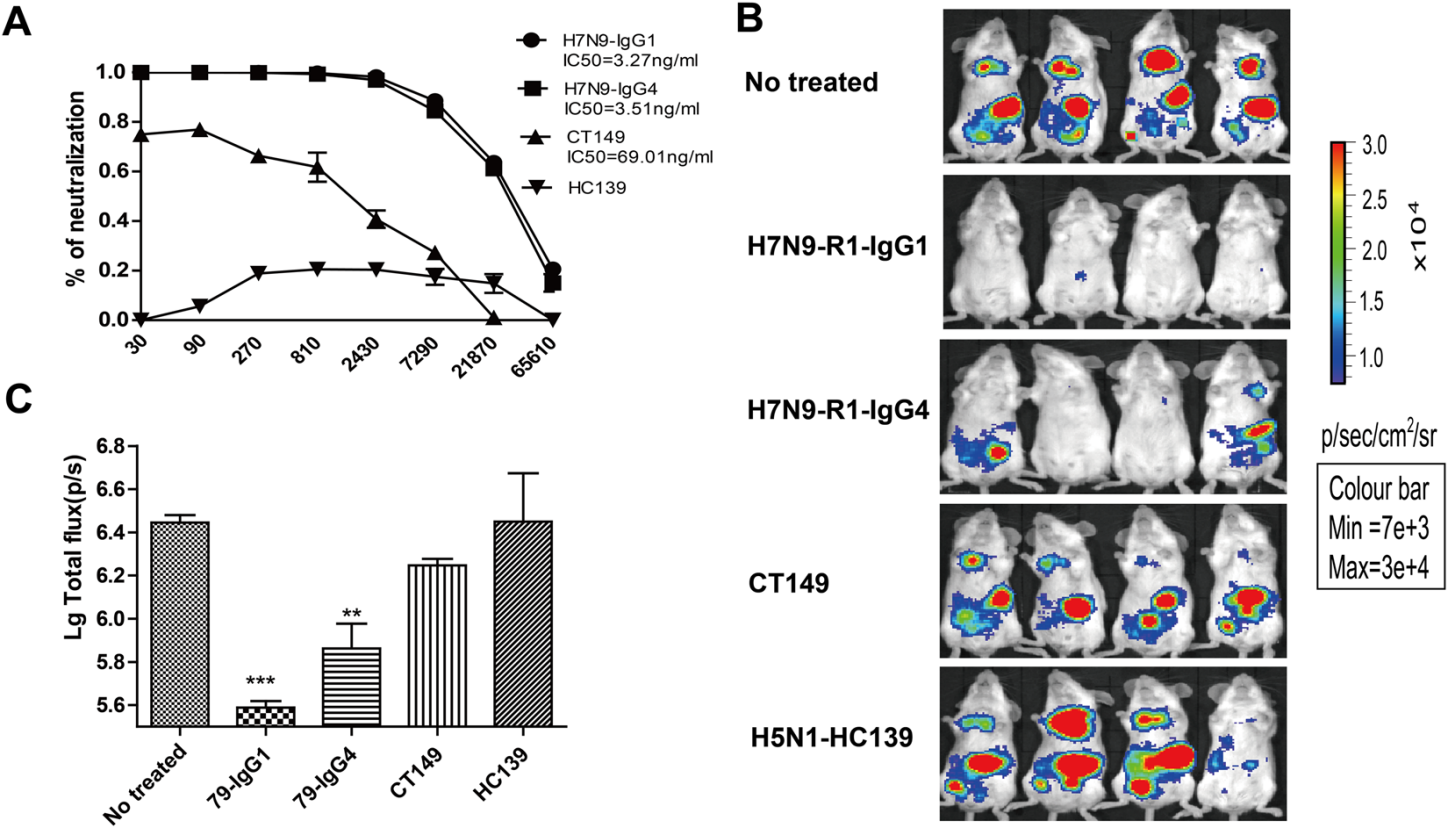
Determination of prophylactic effects of MAbs. Panel (A) Detection of antibody titer of four MAbs by *in vitro* PBNA assay. Panel (B) Bioluminescence imaging of mice treated with MAbs on day 4 post infection. Five- to six-week-old Balb/c mice were intravenously injected with 150 μg of the tested MAbs, followed by intraperitoneal infection with 5.62 × 10^6^ TCID_50_ H7N9-Fluc virus 6 hours later. Panel (C) Analysis of photon flux of group between un-treated and treated groups. The results represents mean ± s.e.m. (n = 4). Statistical analysis was conducted using one-way analysis of variance (ANOVA). ** represents p < 0.01 and *** denotes p < 0.001.

Using PBNA, we tested two H7N9-specific MAbs (H7N9-R1-IgG1 and H7N9-R1-IgG4), one H5N1specific MAb (HC139) and one broadly reactive MAb (CT149)^16^. As shown Fig. 5A, the two H7N9 MAbs exhibited potent inhibitory activity at 3.27 and 3.51 ng/ml (IC50), respectively, while the broadly reactive MAb (CT149) could also inhibit H7N9 replication but at a higher concentration (IC50 of 69.01 ng/ml)^16^. As expected, HC139, the H5N1 specific MAb, displayed no inhibitory activity against pseudotyped H7N9 replication.

To confirm *in vitro* observations, we evaluated inhibitory activities of these MAbs by *in vivo* PBNA assay. To this end, Balb/c mice were passively injected with the MAbs intravenously before challenged with H7N9 pseudovirus 6 hours later. As shown in Fig. 5B and C, both H7N9-specific MAb demonstrated strong inhibitory activities (p < 0.01), while the broadly reactive MAb moderately inhibited viral replication and the H5N1 specific MAb had no inhibitory activity at all. The analyses of photon flux reduction revealed that the two H7N9 specific MAbs afforded 86% and 71% protection, respectively, and the broadly reactive MAb provided 40% protection whereas H5N1 specific MAb failed to provide any level of protection (data not shown). Collectively, these data indicated that the pseudovirus-based *in vitro* and *in vivo* assays could be used for the evaluation of antiviral agents against H7N9.

## Discussion

Pseudovirus has been extensively explored in virus research, antiviral screening and vaccine evaluation^17,18^. Several lines of evidences prompted us to develop H7N9 pseudovirus. Firstly, H7N9 virus can only be handled in BSL3 laboratories, making it difficult for most researchers to conduct antiviral research or vaccine evaluation. Secondly, HI, the traditional assay to assess the immunogenicity of influenza vaccines, has inherent limitations. Specifically, HI only measures the ability of antibodies in inhibiting influenza virus-induced hemagglutination of animal red blood cells; furthermore, the assay is not sensitive for the evaluation of certain influenza vaccines^19^. Thirdly, although MN is thought to be a highly sensitive and specific assay for vaccine evaluation as it quantitatively detects virus-specific neutralizing antibodies in human and animal sera, live wt influenza virus is still needed in the assay^20^; moreover, the MN assay is a relatively complicated procedure with marked variations in results. Fourthly, *in vivo* evaluation of antivirals involves monitoring survival rate of the animals following lethal challenges with the wt viruses. As such, it would be more practical to employ pseudovirus in animal studies in place of wt virus, particularly high pathogenic avian influenza viruses.

To develop H7N9 pseudovirus-based assays, we must first overcome the hurdle that most pseudovirus production systems generate much lower yield of virus than that with the wt virus cultures. Here, we used the backbone plasmid pSG3.Δenv-FlucΔnef rather than pNL4-3-Luc.R.E to package the pseudovirus, resulting more than 100- fold increases in virus yield. The increased titer of H7N9-Fluc pseudovirus allowed us to quantitatively conducted *in vitro* and *in vivo* analyses of virus. We found the results generated by PBNA were largely in agreement with those by either HI or MN (R > 0.8, p < 0.0001). Yet, the GMT values obtained by PBNA and MN were higher than those by HI, indicating that PBNA and MN are measuring similar antibody activities. Given that antibody activities analyzed by MN are thought to be more relevant than those measured by HI as immunological correlates, the use of PBNA could be another ideal alternative tool to evaluate immune responses elicited by influenza vaccines.

The high-titer pseudotyped H7N9 virus also allowed us to develop an animal model in which H7N9 could be visually observed *in vivo*. Such real-time viral detection could be of use to study biodistribution of the virus and quantitatively evaluate vaccine or antiviral agents. Furthermore, it takes only 4 days to determine the effects of antiviral agents using the imaging model in BSL-2 labs whereas it needs over a week to conduct animal studies using wt virus in BSL-3 labs. Although the results generated with the pseudovirus system have not been directly validated with animals studies with live wt virus, our results were consistent with previously reported work in which wt virus was used. Specifically, the broadly-reactive antibody CT149 was reported to protect mice from H7N9 infection with ED50 at 10.9 mg kg^−1 ^^16^, while in our study, administration of 8~10 mg kg^−1^ CT149 afforded 40% protection. The results suggest that our pseudovirus system could be used to evaluate the efficacy of mAb.

It is of note that we were unable to detect fluorescence signal following i.n. administration of the pseudovirus in the animals. This is likely due to the inability of the pseudovirus to replicate. Such inherent limitation of pseudovirus could potentially impede its application to studying viral pathogenesis in place of wt virus. However, the *in vitro* and *in vivo* PBNA assays presented in this communication should be of great value in facilitating evaluation of vaccine and antiviral agents against H7N9.

## Material and Method

### Cell lines and antibodies

All cell lines were maintained at 37 °C in a humidified incubator with 5% CO_2_. Unless specified, the culture media were Dulbecco’s modified Eagle’s medium (HyClone, South Logan, UT) containing 10% fetal bovine serum (Gibco, Carlsbad, CA, USA) and 1% penicillin-streptomycin (HyClone). 293FT were obtained from Invitrogen, Carlsbad, CA; Vero, A549 and MDCK cells were purchased from ATCC, Manassas, VA.

The two H7N9-specific MAbs (H7N9-R1-IgG1 and H7N9-R1-IgG4) and the H5N1-specific antibody (HC139) were obtained from the Sinobiological Inc., Beijing, China. MAb CT149 was kindly provided by Institute of Microbiology, Chinese Academy of Sciences, Beijing, China.

### Serum sample

Five serum panels were used in our study. Seventy-five serum samples were obtained from children under the age of 2; 11 serum samples were isolated from patients recovered from H7N9 infection (Beijing Institute of Basic Medical Sciences, Beijing, China); 100 serum samples derived from healthy donors of different age groups were obtained from Beijing blood center; prior exposure to influenza A virus in these donors were unknown; 40 pairs of serum samples were collected from human subjects before and 6 weeks after trivalent influenza vaccination; 339 serum samples were collected from patients enrolled in a phase I H7N9 clinical trial, with ID CTR20160688 registered at chinadrugtrials.gov.cn. These samples were obtained from Hualan Biological Engineering, Inc., Henan, China. Healthy volunteers were vaccinated twice intramuscularly on days 0 and 21. Of the 339 serum samples, 99, 115 and 125 were collected before and 3 weeks after each vaccination. This study was carried out in accordance with the “Guidelines for the laboratory management of Biological samples analysis in Drug clinical trial” issued by Chinese Food and Drug Administration in 2011, and approved by the Ethic committee of Henan Provincial Center for Disease Control and Prevention in China. For the collection and use of all above-mentioned samples, written informed consents were obtained from all participants.

### Generation of H7N9-Fluc pseudovirus

pSV1.0-H7, pSV1.0-N9, the plasmids encoding HA and NA of A/Anhui/1/2013(H7N9), were constructed to make H7N9 pseudovirus. 293FT cells were co-transfected with HA plasmid, NA plasmid and HIV backbone plasmid (pSG3.Δenv-FlucΔnef or pNL4-3-Luc.R.E) using transfection reagent Lipofectamine 2000 (Invitrogen) at a ratio of 1:1:2 between three plasmids. Forty-eight hours after transfection, the culture supernatants were collected and centrifuged at 4000 rpm for 10 min before they were finally concentrated using 30KD ultrafiltration centrifuge tube. The samples were aliquoted and stored at −80 °C. All experiments involving pseudovirus were performed in a biosafety level 2 facility at the National Institutes for Food and Drug control, Beijing, China.

### Titration of pseudovirus and pseudotyped virus-based neutralizing assay (PBNA)

The pseudovirus was titrated using a luciferase assay kit obtained from Promega, Madison, WI. 50% tissue culture infectious dose (TCID50) was calculated using the Reed-Muench method as described previously^11,21^. Serial dilutions of pseudovirus by five-fold were made in 96-well culture plates (100 μl per well). Then 100 μl of trypsin-treated MDCK cells were seeded into 96-well plates. After 48 hr incubation at 37 °C, 100 μl of culture supernatants were gently removed and discarded. Subsequently, 100 μl of Bright-Glo luciferase substrates (Promega, Madison, WI) were added into each well. After additional incubation for 2 min at room temperature, 150 μl of the lysates was transferred to 96-well plates for luciferase activity determination using Glomax 96 microplate luminometer (Promega, Madison, WI).

For neutralizing assay, all serum samples were heat-inactivated for 30 min at 56 °C prior to the assay. 5000 TCID50 pseudovirus (RLU value of 10^6^) was incubated with human sera (serially diluted by 3-fold) for 1 h at 37 °C in 96-well flat-bottom culture plates. MDCK cells were added in the 96-well plates and incubated for 48 hours before luciferase assay was performed as described above.

### Micro-neutralization (MN) assay and hemagglutinin-inhibition (HI) test

Both MN and HI were performed using the recombinant A/Anhui/1/2013(H7N9) vaccine strain. The MN assay was conducted as described previously^20,22^. In brief, two-fold serial dilutions of heat-inactivated sera were mixed with an equal volume of virus and then incubated at 37 °C for 1 hr. Subsequently, MDCK cells were added to the virus/sera mixture and incubated in 96-well plates at 37 °C for 20 hr. The rest of the procedure was conducted as described^22^.

The HI assay was conducted as described^23^. Briefly, 0.1 ml sera were treated with 0.4 ml receptor destroying enzymes for 16 h at 37 °C and then incubated with chicken red blood cells overnight at 4 °C to absorb the unspecific inhibition agents. The treated samples were collected for the HI test. The HI titer was determined as the reciprocal of the highest dilution at which hemagglutination was completely inhibited.

### Animal experiment

All the animal studies were conducted in accordance with the guidelines set by the Association for the Assessment and Accreditation of Laboratory Animal Care (AAALAC, Frederick, MD). The study protocol was approved by the Animal Care and Use Committee in National Institute for Food and Drug Control (NIFDC, Beijing, China). Five- to six-week-old female Balb/c, C57BL/6, KM and NIH mice were obtained from the Institute for Laboratory Animal Resources, NIFDC (Beijing, China).

The animals were administered with 5.62 × 10^6^ TCID_50_ of pseudovirus by various routes, i.e. intranasal (IN.), intravenous (IV.), intrapleural (IPL.) or intraperitoneal (IP.). Bioluminescence imaging of the mice were analyzed using Xenogen IVIS200 imaging system (Xenogen Corp.).

### Bioluminescence imaging analysis

Bioluminescent imaging was performed with the IVIS Lumina Series III Imaging System (Xenogen, Baltimore, MD) as described previously^24^. In brief, mice were anaesthetized by i.p injection of pelltobarbitalum natricum (240 mg/kg body weight), followed by i.p injected with substrate, D-luciferin (50 mg/kg body weight). The bioluminescence was detected for each mouse 10 min later. Image software (Caliper Life Sciences, Baltimore, MD) was used to measure the luciferase activities; the signals emitted from different ROIs in the body were measured and presented as the total flux in photons/sec/cm^2^/sr.

### Protection of Balb/c mice by MAbs

Five- to six-week-old female Balb/c mice were intravenously injected with 150 μg of MAbs; 6 hours later, they were infected with 5.62 × 10^6^ TCID_50_ via intraperitoneal route. On day 4, the animals were subjected to bioluminescence imaging analysis. Background (substrate only) was subtracted when inhibition rate of the MAb was calculated

### Statistical analysis

All graphs were generated with the Prism 5.0 software (GraphPad, San Diego, CA). All results were calculated and presented as the means ± standard error of the mean (SEM) obtained from triplicate or tetrad tests. The statistical significance was determined by one-way or two-way analysis of variance (ANOVA). P values of <0.05 were considered as statistical significance. Agreement between PBNA and MN was assessed by Cohen’s kappa statistical analysis.

## Electronic supplementary material

Supplementary

## References

[1] Li, Y. *et al*. Evolving HA and PB2 genes of influenza A (H7N9) viruses in the fifth wave - Increasing threat to both birds and humans? *J Infect* **75**, 184–186, S0163-4453(17)30109-3/j.jinf.2017.04.002 (2017). 10.1016/j.jinf.2017.04.002 28392357

[2] World Health Organization. Influenza at the human-animal interface. http://www.who.int/influenza/human_animal_interface/Influenza_Summary_IRA_HA_interface_09_27_2017.pdf?ua=1 (2017).

[3] Dortmans JC (2013). Adaptation of novel H7N9 influenza A virus to human receptors. Sci Rep.

[4] Hu Z, Jiao X, Liu X (2017). Antibody Immunity Induced by H7N9 Avian Influenza Vaccines: Evaluation Criteria, Affecting Factors, and Implications for Rational Vaccine Design. Front Microbiol.

[5] Pan W (2014). Induction of neutralizing antibodies to influenza A virus H7N9 by inactivated whole virus in mice and nonhuman primates. Antiviral Res.

[6] Ou H (2016). Analysis of the immunogenicity and bioactivities of a split influenza A/H7N9 vaccine mixed with MF59 adjuvant in BALB/c mice. Vaccine.

[7] Chen Z (2015). Human monoclonal antibodies targeting the haemagglutinin glycoprotein can neutralize H7N9 influenza virus. Nat Commun.

[8] Du, L. *et al*. Development of a safe and convenient neutralization assay for rapid screening of influenza HA-specific neutralizing monoclonal antibodies. *Biochem Biophys Res Commun* **397**, 580–585, S0006-291X(10)01094-6/j.bbrc.2010.05.161 (2010). 10.1016/j.bbrc.2010.05.161 PMC7092825 20617558

[9] Qiu C (2013). Safe Pseudovirus-based Assay for Neutralization Antibodies against Influenza A(H7N9) Virus. Emerging Infectious Diseases.

[10] Nefkens, I. *et al*. Hemagglutinin pseudotyped lentiviral particles: characterization of a new method for avian H5N1 influenza sero-diagnosis. *J Clin Virol* **39**, 27–33, S1386-6532(07)00076-5/j.jcv.2007.02.005 (2007). 10.1016/j.jcv.2007.02.005 17409017

[11] Nie J (2017). Development of *in vitro* and *in vivo* rabies virus neutralization assays based on a high-titer pseudovirus system. Sci Rep.

[12] Zhang L (2017). A bioluminescent imaging mouse model for Marburg virus based on a pseudovirus system. Hum Vaccin Immunother.

[13] Qiang L (2017). Antibody-dependent-cellular-cytotoxicity-inducing antibodies significantly affect the post-exposure treatment of Ebola virus infection. Sci Rep.

[14] Xu L (2013). The mouse and ferret models for studying the novel avian-origin human influenza A (H7N9) virus. Virol J.

[15] Belser JA (2013). Pathogenesis and transmission of avian influenza A (H7N9) virus in ferrets and mice. Nature.

[16] Wu Y (2015). A potent broad-spectrum protective human monoclonal antibody crosslinking two haemagglutinin monomers of influenza A virus. Nat Commun.

[17] Garcia JM, Lai JC (2014). Production of influenza pseudotyped lentiviral particles and their use in influenza research and diagnosis: an update. Expert Review of Anticancer Therapy.

[18] Carnell GW, Ferrara F, Grehan K, Thompson CP, Temperton NJ (2015). Pseudotype-based neutralization assays for influenza: a systematic analysis. Front Immunol.

[19] Nogales A, Baker SF, Domm W, Martinez-Sobrido L (2016). Development and applications of single-cycle infectious influenza A virus (sciIAV). Virus Res.

[20] World Health Organization. Serological detection of avian influenza A(H7N9) infections by microneutralization assay. http://www.who.int/influenza/gisrs_laboratory/cnic_serological_diagnosis_microneutralization_a_h7n9.pdf?ua=1 (2013).

[21] Li M (2005). Human immunodeficiency virus type 1 env clones from acute and early subtype B infections for standardized assessments of vaccine-elicited neutralizing antibodies. Journal of Virology.

[22] Rowe T (1999). Detection of Antibody to Avian Influenza A (H5N1) Virus in Human Serum by Using a Combination of Serologic Assays. Journal of Clinical Microbiology.

[23] Smithwick, R. W. Concepts and procedures for laboratory-based influenza surveillance. *Epidemiology* (1982).

[24] Zhu R, Liu Q, Huang W, Yu Y, Wang Y (2014). Comparison of the replication characteristics of vaccinia virus strains Guang 9 and Tian Tan *in vivo* and *in vitro*. Archives of virology.

