# Supplementary material for: Development of in vitro and in vivo neutralization assays based on the pseudotyped H7N9 virus
Source: Sci Rep. 2018 May 31;8:8484. doi: 10.1038/s41598-018-26822-6 (PMC5981435; doi:10.1038/s41598-018-26822-6)
Supplement: Supplementary file 1 — Supplementary [file 41598_2018_26822_MOESM1_ESM.doc]

**Development of *in vitro* and *in vivo* neutralization assays based on the pseudotyped H7N9 virus**

Yabin Tian^1^, Hui Zhao^3^, Qiang Liu^2^, Chuntao Zhang^1^, Jianhui Nie^2^, Weijing Huang^2^, Changgui Li^3^, Xuguang Li^4^, Youchun Wang*^2^

**Supplementary figure .S1**


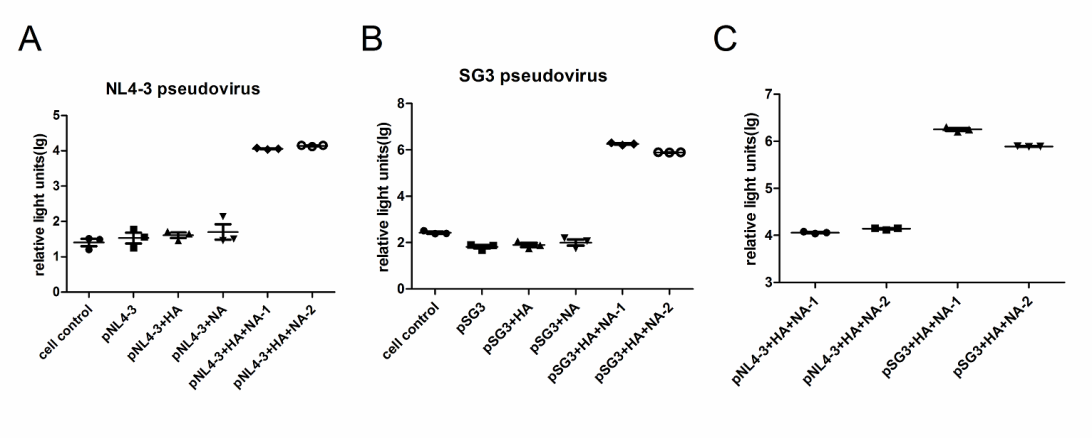


**Figure S1**. Optimation of backbone plasmid and generation of pseudovirus.

Panel A: Generation of pseudovirus based on the pNL4-3-Luc.R.E (4-3) backbone plasmid with different situation.

Panel B: Generation of pseudovirus based on the pSG3.Δenv-FlucΔnef (SG3) plasmid with different situation.

Panel C: Comparison of backbone plasmid based on pNL4-3-Luc.R.E. or pSG3.Δenv-FlucΔnef. 1 and 2 represent different amount of plasmid of packaging pseudovirus (1: 5μg NL4-3/SG3, 2.5μg HA and 2.5μg NA in 6-well plate, 2: 4μg NL4-3/SG3, 2μg HA and 2μg NA in 6-well plate)

**Supplementary figure.S2**

**
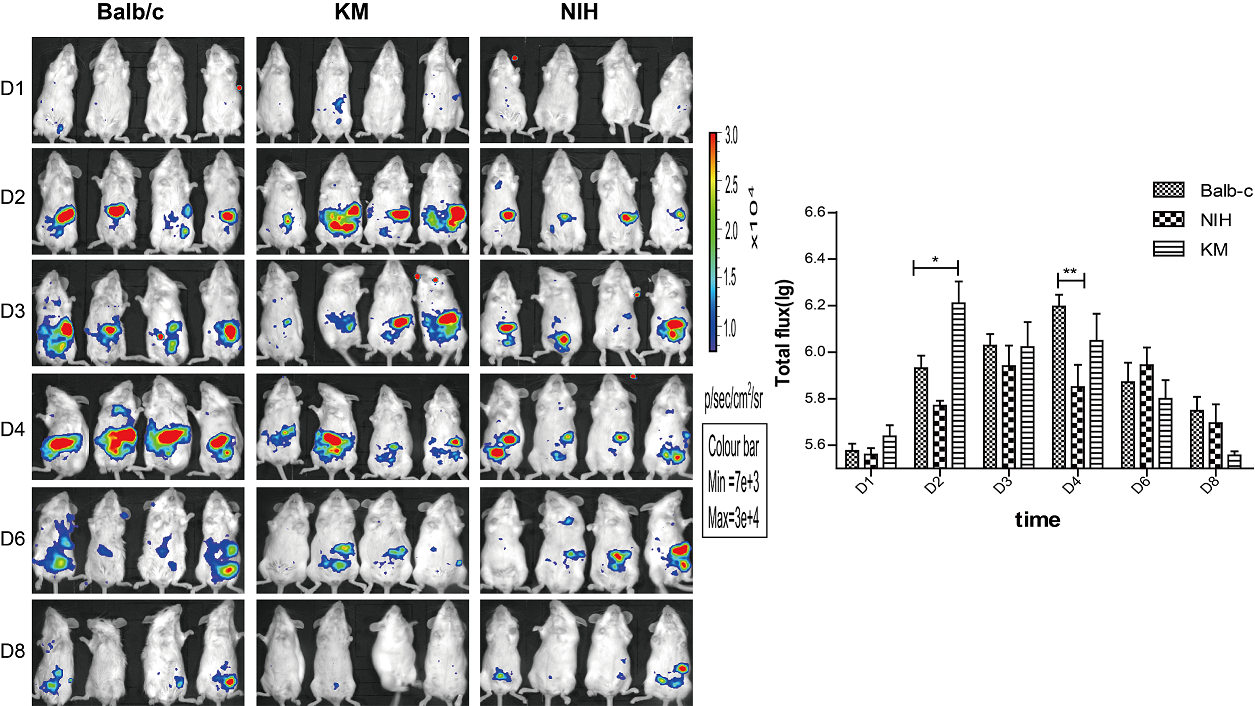
**

**Figure S2**.Different mice infected intraperitoneally with H7N9 pseudovirus （5.62×10^6^TCID_50_ per mouse）were imaged at day1 to day8, Results are shown as mean±s.e.m (n=4/group). Significant effects of signal are for Balb/c in day 4 (Balb/c vs NIH, ***p* < 0.01, two-way ANOVA) and for KM in day 2 (Balb/c vs KM, **p* < 0.05, two-way ANOVA).

**Supplementary figure.S3**


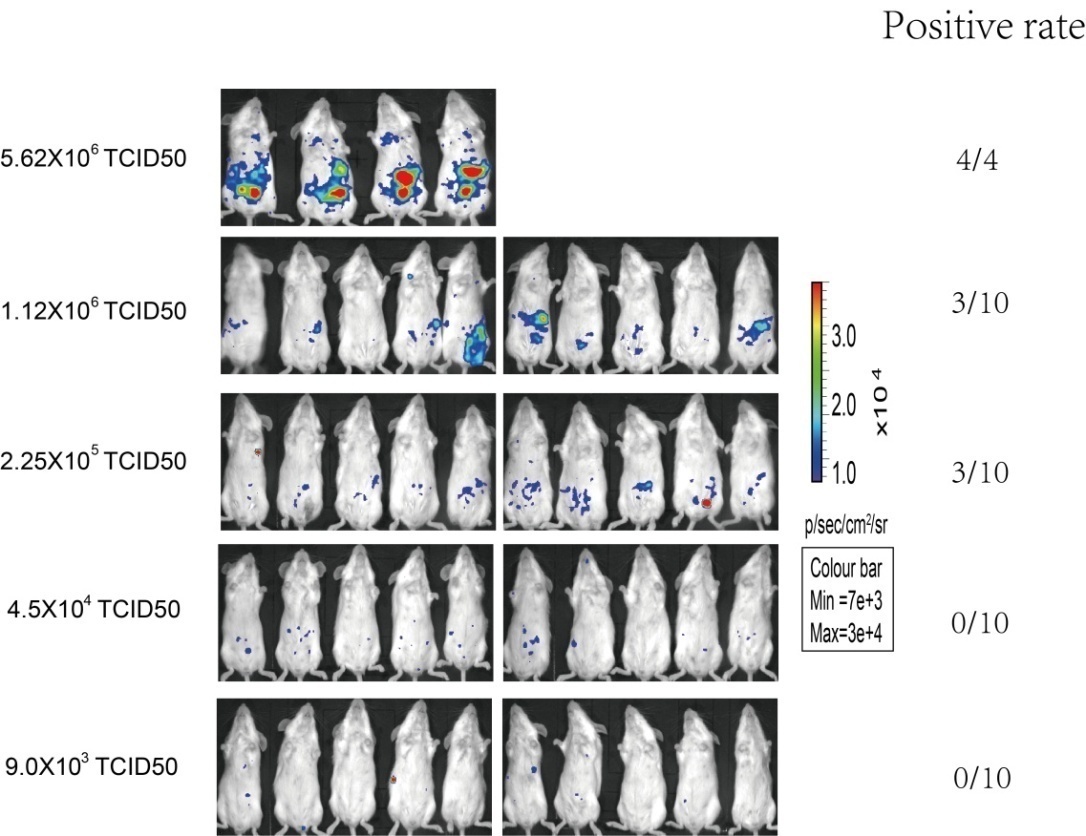


**Figure S3**. Determination of challenge dose. Five- to six-week-old Balb/c mice were inoculated with H7N9 pseudotyped virus from 9 to 5620 ×10^3^TCID_50_ per mouse. The bioluminescence imaging was analyzed on day 4.

**Supplementary Table.S1** Comparison of the geometric mean titers (GMT) and standard deviation obtained from MN, HI and PBNA.

|  | Day0 | | Day21 | | Day42 | |
| --- | --- | --- | --- | --- | --- | --- |
|  | GMT | SD* | GMT | SD* | GMT | SD* |
| MN | 6 | 0.14 | 116 | 1.07 | 271 | 1.21 |
| HI | 5 | 0.00 | 30 | 0.67 | 54 | 0.79 |
| PBNA | 39 | 0.29 | 128 | 0.79 | 226 | 0.89 |

*SD was calculated from log10 data of antibody titre.

**Supplementary Table.S2 Concordance between PBNA and MN.**

|  | | PBNA | | Agreement between PBNA and MN | | |
| --- | --- | --- | --- | --- | --- | --- |
|  |  | Positive | Negative | Sensitivity | Specificity | Kappa coefficient |
| MN | Positive | 142 | 3 | 98% | 92% | 0.89 (95%CI:0.85-0.92) |
|  | Negative | 16 | 178 |  |  |  |
